# Post-20^th^ century near-steady state of Batura Glacier: observational evidence of Karakoram Anomaly

**DOI:** 10.1038/s41598-020-57660-0

**Published:** 2020-01-22

**Authors:** Haifeng Gao, Xiaojuan Zou, Jianfeng Wu, Yinsheng Zhang, Xiaoya Deng, Saulat Hussain, Muhammad Atif Wazir, Guocai Zhu

**Affiliations:** 1 Key Laboratory of Tibetan Environment Changes and Land Surface Processes, Institute of Tibetan Plateau Research, Chinese Academy of Sciences, Beijing, China; 2 Satellite Environment Center, Ministry of Ecology and Environment, Beijing, China; 3 State Key Laboratory of Simulation and Regulation of Water Cycle in River Basin, China Institute of Water Resources and Hydropower Research, Beijing, China; 4 Northwest Institute of Eco-Environment and Resources, Chinese Academy of Sciences, Lanzhou, China

**Keywords:** Cryospheric science, Environmental impact

## Abstract

Stable or marginal mass loss dominating in Karakoram has been reported widely through satellite and ground investigations. This work aimed to verify the variation in glacier mass by collecting ground-based data. By tracking profiles from the first survey by China–Pakistan Batura Glacier Investigation Group in 1974–1975, we revisited Batura Glacier and conducted an updated comparable measurement of the glacier surface elevation and ice thickness of this large valley glacier of Karakoram, in August 2017. Results of ground penetrating radar (GPR) measurement were used to improve the accuracy of an ice thickness distribution model (GlabTop2). The model calculation agreed reasonably with the measurement when the optimal basal shear stress (100 kPa for clean ice to 140 kPa for heavy debris cover) and shape factor (0.9) were used. We then used a glacier bed topographies map to calculate the ice flux. By subtracting the glacier surface topographies from the remote-sensing measurements, we observed a marginal thinning in Batura during 2000–2016, with a rate of variation in glacier surface elevation of −0.12 ± 0.27 m a^−1^. It indicated that the mass gain in the accumulation area nearly compensated the mass loss in the ablation area. In addition, both ground and satellite remote measurement reveal a steady rate of decrease in surface of the Batura tongue, implying an absence of significant variation during the past 40 years. Moreover, the mass conservation equation was applied to the Batura tongue, in combination with surface elevation variation and ice flux evolution. The tongue-averaged mass balance diminished by more than half from the 1970s to the 2010s. In summary, we inferred a near-steady state of Batura Glacier post 2000 based on the above-mentioned evidence of “Karakoram Anomaly”.

## Introduction

Glaciers are important water resources and exceptionally sensitive indicators of climate change. Glacier melt water plays an important role in the Indus basin^1^ owing to its extensive upstream and glaciated area. Millions of humans rely on the meltwater from the Upper Indus Basin (UIB). The glacial response to climate change in this region has important ramifications for future water availability from UIB.

UIB is composed of the Hindukush, Karakoram, and Himalayan mountains (HKH). It has the highest concentration of glaciers outside the polar regions^2^. The glaciers cover 12,705 km^2^. Of this, over 70% is in the Karakoram region^3^. However, there is high variability in the glacier ice volume estimates of the Karakoram region, ranging from 1683 to 2827 km^3^ by different approaches^4^. Measured ice thickness data can be applied to validate and increase the accuracy of volume estimation at the regional scale^5,6^. Measurements of the ice thickness in High Mountain Asia are few compared with those of the glacier amount. In addition, glaciers here have displayed an unusual behavior during the past decades: an absence of significant area variations^7^, rather, frequent advances and surge activities^8,9^. Stable or marginal mass loss dominate in Karakoram (“Karakoram Anomaly”), whereas glaciers worldwide are retreating^10,11^. The major conclusion had originated from remotely sensed glacier surface elevation variation of either points (satellite laser altimetry) or surfaces (digital elevation models)^12–16^. Because of the rugged terrain and the challenges involved in field surveys, long-term observation data on HKH is sparse. Thus, the observed evidences are crucial for glacier mass change study.

Surface mass balance is vital to the survival of a glacier. It is strongly related to climate change. It cannot be directly measured regionally by remote-sensing methods^17^. Rather, it could be obtained from either ablation stake measurement or using equation methods. The equation of mass conservation, applied to a glacier tongue, indicates two major processes potentially driving the glacier surface elevation change^17,18^: (i) ablation at the glacier surface and (ii) net ice flux from upstream to downstream. Meanwhile, accurate data on ice thickness is important for ice flux calculation.

Although arduous field monitoring on glacier change is necessary, it can be expensive. Therefore, representation of the selected glaciers with limited resources is highly important. Research on mountain glaciers focuses mostly on medium-sized and large valley glaciers. A time lag generally occurs between a change in climatic conditions and the resulting glacier change^19^. Compared to small glaciers, large glaciers respond more gradually and are steadier to perturbations in climate^20,21^. Results from short-term observations cannot be in accordance with the real climate change. The absolute change of larger glaciers contributes more significantly to the hydrological regime owing to the larger size^22^. Whereas the use of remote sensing data yields relatively larger errors for glaciers of small size^23^, fewer problems are encountered for larger glaciers, wherein larger changes occur^24^. Accordingly, we consider the results from large glaciers research to be more reliable. Batura Glacier, one of the largest valley-type glaciers in the Hunza basin of UIB, is located in northwest Karakoram. Detailed mapping and ground-based investigations have been carried out to detect its effects on the Karakoram Highway during 1974–1975 and the past few years by different research teams^25–27^. The glacier area has reduced from ~285 km^2^ in 1974^27^ to ~243.5 km^2^ at present^28^. Debris covers ~27% of the glacier area^28^, and its thickness in the part below 3000 m a.s.l. exceeds 50 cm according to our field survey. Because of its important geographical position and valuable historical records, Batura Glacier would be a more appropriate representative for studying glacier changes in the Karakoram region.

**Figure 1:**
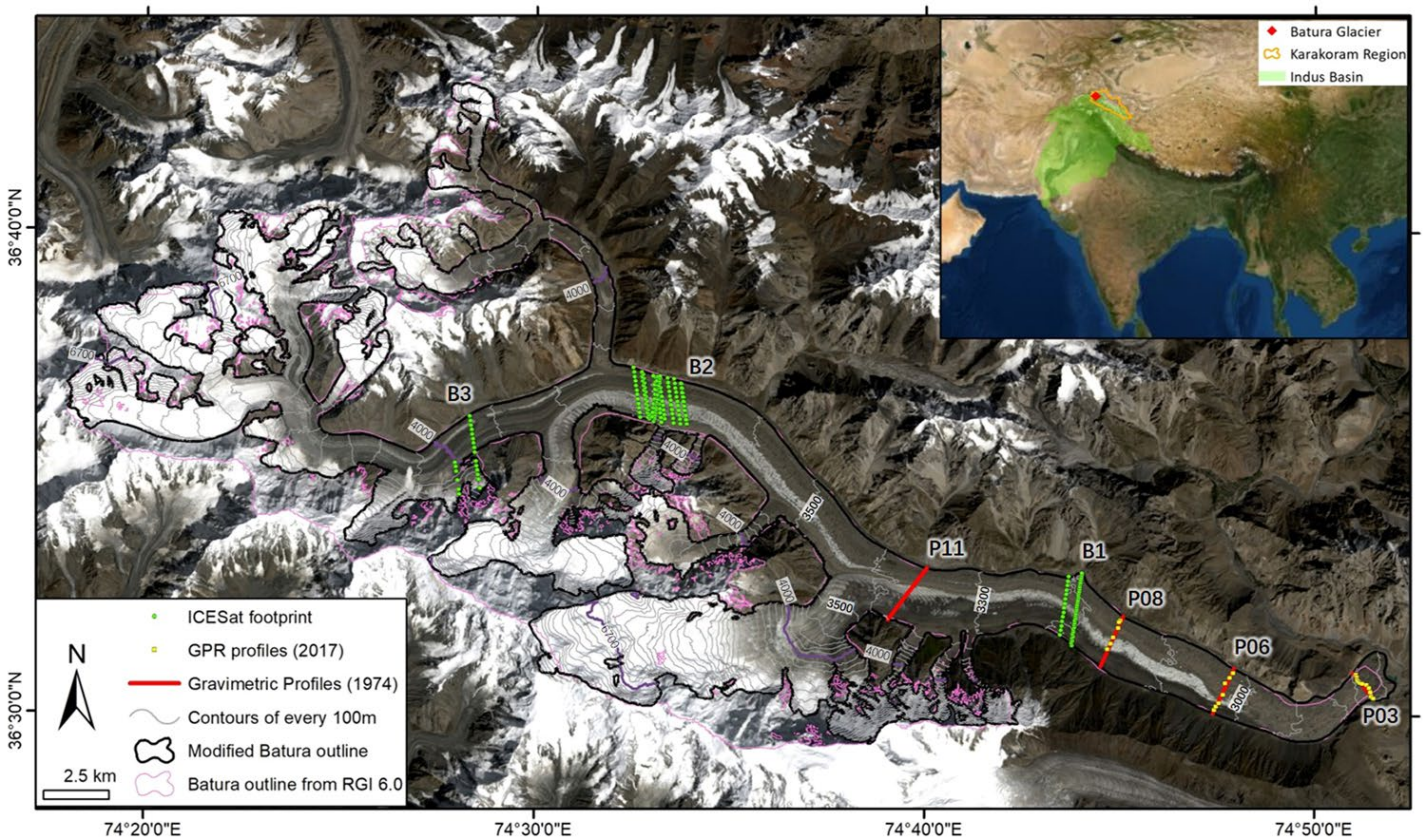
Topography of Batura Glacier with ice thickness measurement and ICESat footprint. This map was generated from open-source datasets using ArcGIS version 10.3 (https://www.esri.com/zh-cn/arcgis/products/arcgis-pro/overview). The background is a true color image derived from the Landsat scene on September 9, 2017 (https://earthexplorer.usgs.gov/). Each contour interval for surface elevation is 100 m. The red curves indicate profiles of gravimetric measurements of ice thickness in 1974. The yellow dots indicate GPR measurements of ice thickness in 2017. The green dots indicate ICESat footprints with different elevation range. The black thick line is the glacier outline of Batura that we used in this study, modified from the newest Glacier Inventory of Pakistan. The pink line is from Randolph Glacier Inventory (RGI) 6.0. The upper right map shows the location of Batura Glacier, the Karakoram Mountain region, and Indus Basin. The background of the inset map is from Google Earth satellite images. The Karakoram boundary is based on the work by Rankl *et al*.^8^. The watershed of Indus is extracted in ArcGIS by using an SRTM dataset provided by the CGIAR Consortium for Spatial Information (http://srtm.csi.cgiar.org/).

To supplement the glacial database of ice thickness in the Karakoram region, we propose a fresh comparable measurement of ice thickness in Batura Glacier (Fig. 1) using ground penetration radar (GPR). The measurement data were used to validate the results of a simulation of an ice thickness distribution model (GlabTop2). The first goal of the present study was to improve the accuracy of calculation of ice thickness by the model and thereby, precisely estimate the ice volume of Batura Glacier. Thereafter, we revisited historical records and satellite data and investigated glacier changes in surface elevation during the past 40 years. We could reliably infer the robust bedrock topographies (necessary information for ice flux calculation) with the accurate ice thickness values^29^ determined in fulfilment of the first goal. Here, a remaining unknown variable of the mass conservation equation was determined from the other two by selecting appropriate known variables. The second goal of this study was to investigate the variations in tongue-averaged surface mass balance of Batura with observational evidence.

## Results

### Ice thickness and volume

**Figure 2:**
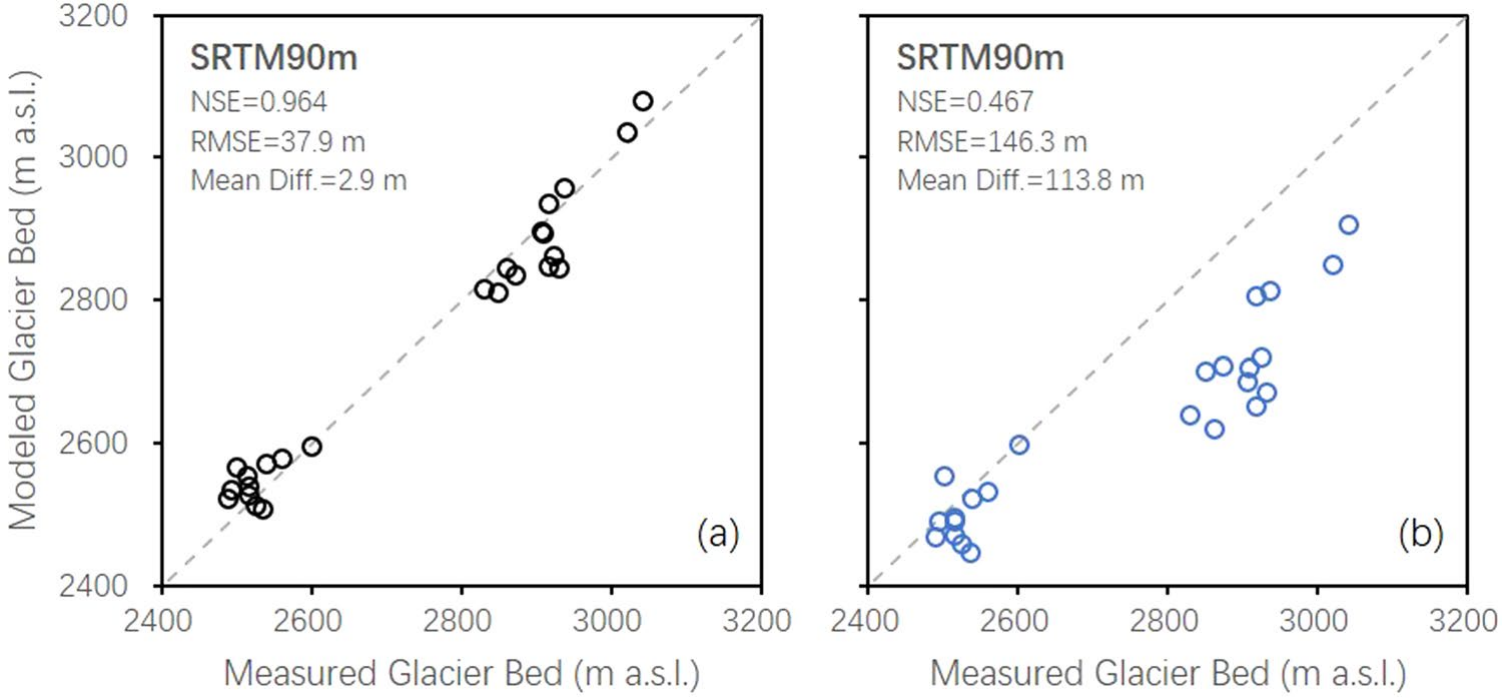
Measured versus modeled glacier bed position results. The parameterization of the model is from this study (**a**) and Frey *et al*. (**b**), respectively.

**Figure 3:**
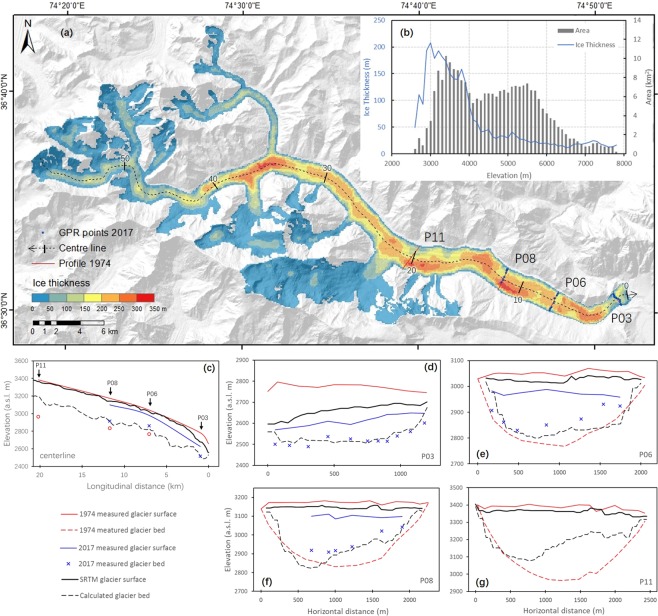
Ice thickness of Batura Glacier. (**a**) Shows the ice thickness distribution of the whole glacier at 50 m intervals. This map was generated by the open-source datasets using ArcGIS version 10.3. (https://www.esri.com/zh-cn/arcgis/products/arcgis-pro/overview). The hill-shaded background in this figure has been generated by using the SRTM DEM (http://srtm.csi.cgiar.org/). The mean ice thickness change with altitude is shown in (**b**). Profiles with measured (blue and red curves) and calculated (black curve) ice thickness are shown in insets (**c**–**g**).

The GlabTop2 approach is designed based on a fundamental consideration of glacier flow dynamics. It was introduced by Frey in 2014^4^. The thickness of ice appears to be governed mainly by its surface slope under the perfect-plasticity assumption^30^. It enables accurate ice thickness calculation with the surface slope (*α*) and two core parameters (basal shear stress (*τ*) and shape factor (*f*)). Specifically, we compared values of glacier bed position simulated by the GlabTop2 model with GPR measurement data. Thereby, we determined the optimal basal shear stress (*τ* = 140 kPa for area covered with thick debris; *τ* = 100 kPa for the remaining parts) and shape factor (*f* = 0.9). Figure 2a illustrates the remarkable agreement between the measurement and simulation results of this study. The higher Nash–Sutcliffe efficiency (NSE) and root mean square error (RMSE) indicates that the results of the present study are better than those of Frey^4^ (Fig. 2). This is likely to be owing to the improved parameterization of the model through ingestion of calibrated shape factor, improved basal shear stress, or both. A relative uncertainty of 25.4%, i.e., ±37.9 m to the average measured thickness of 149.4 m, is considered for the ice thickness calculation. Figure 3 shows the distribution of the simulated ice thickness in Batura Glacier. The mean ice thickness is over 150 m. This is observed below 4000 m a.s.l of the trunk part. Owing to the flat glacier surface, the ice thickness increases as the elevation reaches between 3100 and 3800 m a.s.l. Meanwhile, it decreases dramatically as the slope steepness increases when the elevation is over 4000 m a.s.l. The profiles of both measured and modeled results are plotted in Fig. 3c–g. The uncertainty in the estimation of glacier volume depends on the error in the ice thickness calculation and has been quantified using the error propagation method^31^. According to our calculation, the total ice volume of Batura in 2000 is estimated as 18.88 ± 4.79 km^3^ (Table 1). The ice volume of Batura below 4000 m a.s.l. is 75.5% of the total ice volume, whereas it covers approximately 37.4% of the total glaciated area. The ice volume of Batura was 18.43 ± 4.90 km^3^ in 2016 considering the ice surface elevation change during 2000–2016 (next section).

**Table 1:** Glacier area and volume of each elevation band.

Elevation range (m)	Glacier Area (A/km^2^)	Ice Volume in 2000 (V/km^3^)	Mean dh/dt* (m a^−1^)	Ice Volume Change* (ΔV/km^3^)	Ice Volume in 2016 (V/km^3^)
2570–4000	91.20	14.26 ± 3.62	−0.57 ± 0.18	−0.83 ± 0.26	13.43 ± 3.63
4001–6700	142.80	4.44 ± 1.13	0.17 ± 0.35	0.39 ± 0.80	4.83 ± 1.38
6701–7795	9.53	0.18 ± 0.05	−0.06 ± 0.39	−0.01 ± 0.06	0.17 ± 0.08
Total	243.53	18.88 ± 4.79	−0.12 ± 0.27	−0.45 ± 1.05	18.43 ± 4.90

Note: *The value was calculated in 2000 and 2016.

### Glacier surface elevation change

**Figure 4:**
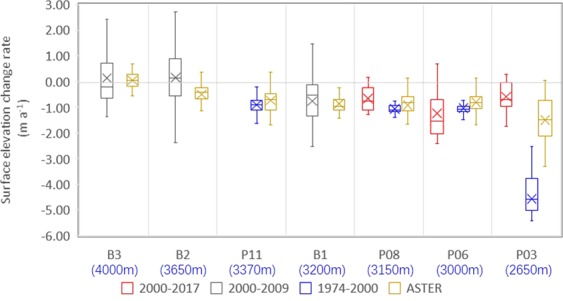
Boxplot of rate of change in Batura Glacier surface elevation at different sections: derived from the difference between field survey results of 1974 and of SRTM DEM of 2000 (Blue box), ICESat data of 2000–2009 (Grey box)^33^, the difference between SRTM DEM of 2000 (http://srtm.csi.cgiar.org) and field survey of 2017 (Red box), and ASTER DEMs of 2000 and 2016 (Yellow box)^32^. The band inside the box is the median value, and the cross sign represents the mean of the data. The altitude of each profile is presented under the profile name as a blue number in brackets.

The rate of surface elevation change of Batura Glacier is determined from point measurements recordeds between 1974 and 2017 (Fig. 4). The thinning of the ice decreased progressively with increasing altitude. The rate of glacier thinning decreased non-significantly over time. However, the most significant thinning occurred at the glacier snout. A dramatic thinning was observed at P03 (−4.58 m a^−1^) from 1974 to 2000. It had thinned at significantly higher rate than the other three upper profiles over the same time period. Meanwhile, the measured thinning rate (−0.59 m a^−1^) at P03 during 2000–2017 is less negative than the rate during the previous period.

**Figure 5:**
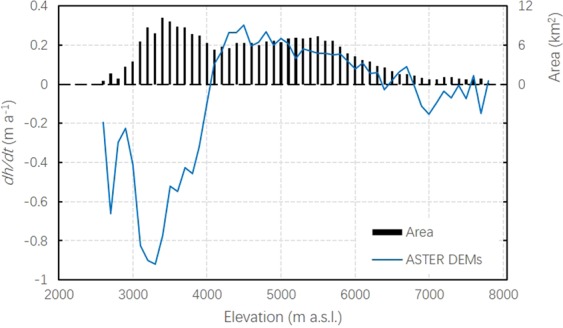
Mean surface elevation change rate (blue curve) for entire Batura. It is derived from ASTER DEMS of 2000–2016, which was extracted by the work of Brun *et al*.^32^, in combination with glacier area distribution along the altitude.

We have also determined the changes in ice surface elevation using ASTER DEM data^32^ (Fig. 5) of 2000 and 2016. A reduced elevation change is visible below 4000 m a.s.l., and the change varied widely. Above 4000 m a.s.l., the glacier surface elevation change rate increased to 0.21 m a^−1^. Hence, the Batura Glacier could be divided into three zones according to the rate of surface elevation change (Table 1): below 4000 m, above 6700 m, and the intervening zone. Over half of the mountain glacier area is covered by the thickening zone. The rate of change in mean surface elevation of Batura Glacier from 2000 to 2016 was −0.12 ± 0.27 m a^−1^ (equivalent to −0.45 ± 1.05 km^3^ of ice). The observed reduction is marginal. Moreover, the negative mass balance change appeared to be less intensive than those in the Himalayan region^32–34^. Meanwhile, the mean rates of change in glacier surface were initially positive above 4000 m and 3650 m according to ASTER DEM and the ICESat data, respectively. This indicates that the surface mass balance should be positive at these altitudes according to the mass conservation equation (i.e., the equilibrium line is lower than these altitudes). Whereas, the annual 0 °C isotherm was estimated at an elevation of approximately 4200 m a.s.l. in 1974^27^. It reflects that the equilibrium line has moved downward post 2000.

### Surface mass balance of lower batura

The tongue-averaged mass balances below each flux gate for two periods (Table 2) are estimated from the emergence velocity and surface elevation change, considering the calibrated glacier bed topography. The area-weighted mass balances of the later period were less negative than those in the 1970s. However, an increase in mass balance post 2000 indicates that the glacier ice volume has been increasingly positive. For example, the average surface mass balance in the section P11–08 (−5.32 ± 0.78 m a^−1^ of ice in 1974–1975) has been increased substantially to −1.81 ± 1.29 m a^−1^ in 2007–2011. The tongue-averaged mass balance below profile P11 diminished from −4.84 to −2.25 m a^−1^ from 1970s to 2010s.

**Table 2:** Average surface elevation change (*Δh*), emergence velocity (*v*_*e*_), and surface mass balance (*B*) below each profile at the lower part of the glacier during 1974–1975 and 2007–2011. The mass balance and glacier surface velocity of the early period was measured by field survey^27^. The ice surface elevation change and emergence velocity of 2010s are computed using the velocity values derived by Rankl *et al.*^8^ and topography by Brun *et al.*^32^, respectively. All the data are in m a^−1^ of ice.

Section	Period	*Δh*	*Δv*_*e*_	*B*
P11–08	1974–1975	−4.78 ± 0.11^*^	0.54 ± 0.07	−5.32 ± 0.78
	2007–2011	−0.89 ± 0.50	0.93 ± 1.19	−1.81 ± 1.29^*^
P08-06	1974–1975	−2.26 ± 0.12^*^	2.61 ± 0.09	−4.87 ± 0.75
	2007–2011	−0.89 ± 0.55	1.41 ± 0.86	−2.30 ± 1.02^*^
P06-terminal	1974–1975	−1.75 ± 0.08^*^	2.23 ± 0.04	−3.98 ± 0.68
	2007–2011	−0.42 ± 0.59	2.58 ± 0.42	−3.00 ± 0.73^*^

Note: *The value is calculated according to Eq. (1).

## Discussion

### Improvement of ice thickness calculation

Basal shear stress and shape factor are the key parameters in thickness calculation^4,31,35^, which was optimized by measured ice thickness. Several studies on mountain valley glaciers have revealed that the shear stress on the glacier’s bed ranges from 30 to 160 kPa^36–38^. A constant basal shear stress value of 100 kPa^39,40^ and other adjusted values have been used in individual glaciers^30^ and regions^41,42^. In general, 150 kPa was used empirically as the maximum basal shear stress in estimating slope-dependent thickness over glaciers of the Karakoram region^4,42,43^. However, owing primarily to the large scale and gentle slope in the trunk of Batura, valley walls support part of the glacier’s weight. The basal shear stress here would be less than that for an infinitely wide and steep channel. In addition, the heavy debris-covered glacier section has higher hydrostatic pressure at the glacier bottom because of the large difference in density between ice and debris. This pressure has been causing a gradual increase in the basal shear stress. Debris impact cannot be omitted in ice thickness calculation. It has been reported that the shape factors related to the lateral drag predominated because of the friction of the valley walls and the general form of the glacier cross-section^31,43^. It depends specifically on the ratio of its half-width to the centerline thickness by Nye^44^. The uncertainty in the modeled ice thickness because of a variation in *f* from 0.7 to 0.9 attains ±12.5%^43^. The width-to-depth ratio of the trunk part here is more than four and corresponds to a larger shape factor (*f* > 0.8), according to the *f* values through parabolic cross-sections (cf. Table 11.3 in *The Physics of Glaciers*^45^).

The original parameterization (*τ*_*max*_ = 150 kPa and *f* = 0.8) in a previous study^4^ may result in overestimation of the ice volume of Karakoram glaciers (Fig. 2). Based on GPR observation, we have determined that the maximum basal shear stress of 100 kPa and shape factor of 0.9 are more suitable for ice thickness calculation in the upper part of Batura, where the debris thickness is less and could be omitted. Meanwhile, a larger *τ* of 140 kPa would be better for the lower part of the glacier, which is under a thick debris layer. The difference between GPR measurements and ground-based gravimetric analysis for measuring ice thickness of glaciers is apparent. As a standard tool in glacier ice thickness measurement at present, GPR has established its suitability for glaciological use with less uncertainty than gravimetric analysis^46,47^. The uncertainties associated with the status and fate of Karakoram glaciers are attributed mainly to deficient information^48^, particularly ice thickness measurement. Measuring the thickness of selected glaciers for mapping subglacial topography, calculating ice volumes and ice flux are therefore required more from recent developed various calibrating methods. The results of this study highlight the potential for more accurate estimation of fresh water reserves stored in the UIB glaciers and their potential contribution to sea-level rise.

### Observed evidence of stable batura glacier post 2000

It is well established from previous studies that glaciers in Karakoram have been relatively stable or re-advanced^11,14,33,34,49^ compared to rapidly retreating glaciers in the Hindukush and Himalayan regions during the past decades^32–34^. The most recent observations of the stable status of Batura Glacier, a large glacier in west Karakoram, established that the phenomenon of “Karakoram Anomaly” exists. Our observations are analogous to this fact in at least three aspects. First, only slight reduction in the glacier surface elevation change was observed during 2000–2016. The mean surface elevation change rate of Batura is −0.12 m a^−1^ (with an uncertainty of ±0.27 m a^−1^). This is significantly less than that reported previously in the Himalayan region^10^. The elevation of approximately 4000 m a.s.l. denotes the transition boundary between the upstream region of thickening (accumulation area) and the downstream region of thinning (ablation area) (Fig. 5). The upstream thickening and downstream thinning causes the glacier surface to steepen, increasing the local driving stresses^50^. A negative budget was observed in the ablation area post 2000 (Fig. 4), which is consistent with previous observations of 1999–2009^12^. Meanwhile, the mass gain in the accumulation area marginally covered the mass loss of the middle and downstream parts, resulting in an overall marginal negative mass balance (Table 1).

**Figure 6:**
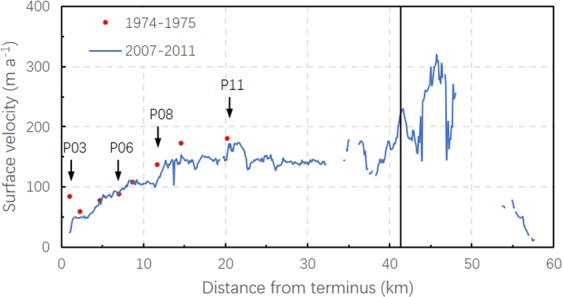
Centerline velocity of Batura. The black line is the dynamic balance line, which represents the driving stress. The colors correspond to surface velocity values from different data sources: the blue line corresponds to values from SAR satellite imagery^8^ and the red spots to those from field observation^27^.

Secondly, the surface decreasing rates of Batura tongues are stable. There has been no significant shift during the past 40 years (Fig. 4). A remarkable meltdown and retreat was observed near the terminus during 1974–2000. The retreat in the early 1980s is underscored by the work of Zhang *et al*.^26^. The frontal ice cliff declined in slope and was covered with debris and vegetation^51^. From the centerline velocity profile (Fig. 6) derived from results of Rankl *et al*.^8^, no surge front formed in the ablation zone between 2007 and 2011. The values during 1974–1975 behaved similarly, increasing only in speed along the terminus of 1980s. Low surface velocities (<200 m/a^−1^) were maintained in the ablation area, and they decreased markedly near the terminus. However, the surface velocity of Batura Glacier did not slowdown concomitant with the thinning of the ice^26,52^. In addition, it was observed that from 1992 to 2007, its terminus shifted forward by almost 100 m^53^. The long-term equilibrium state indicates that Batura is now in a period of relative stagnation.

Remarkably, the ablation of lower Batura had been demonstrated to have decreased based on mass conservation calculation (Table 2). The calculated surface mass balance (i.e., ablation) decreased over time in the entire observed sections of the lower part of Batura. The changes in surface mass balance are directly related to climate fluctuations. The decreasing ablation coincides well with the significant cooling in the glacier melt season^54^. The mass balance observations of Karakoram mountain glaciers are few, particularly in the heavily debris-covered parts. If bedrock topography had been measured or simulated, the geometric terms (thinning rate and ice flux) could potentially have been derived for large glacier tongues, using remote sensing techniques^17^. This strategy would be particularly relevant to the determination of tongue-average surface mass balance change in glaciers about which observational data are limited.

## Methods

### Processing scheme

**Figure 7:**
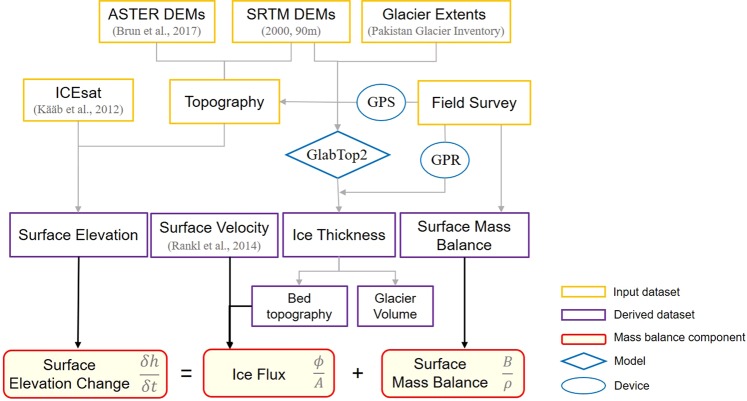
Datasets and processing scheme used in this study.

The datasets and process flow of this study are presented in Fig. 7. A slope-dependent estimation model (GlabTop2) was used to calculate the ice thickness of Batura Glacier. The calculation was validated by field measurement using ground penetration radar (GPR). The bed topography and glacier volume were obtained accordingly (Fig. 3). The former was then used with the surface velocity to determine the ice flux (emergence velocity). Meanwhile, multiperiod changes in glacier surface elevation were analyzed using data from multiple datasets. These changes are presented as profiles (Fig. 4) and along the elevation gradient (Fig. 5), respectively. Thereafter, the glacier surface mass balance of Batura (Table 2) was estimated according to the values of surface elevation change and ice flux by using Eq. (1).

### Data acquisition

#### Glacier boundary

The outline of Batura Glacier was obtained from Glacier Inventory of Pakistan (GIP)^28^. GIP was determined from 24 Landsat scenes between 2013 and 2015 and has the most recent separate boundaries of both clean ice and debris-covered areas. We extracted Batura Glacier and modified it visually along the margin of the glacier’s tongue with Landsat and Google Earth images, as well as data from a field investigation in August 2017. In addition, it is compared with the boundaries from Randolph Glacier Inventory (RGI) 6.0 (https://www.glims.org/RGI/rgi60_dl.html) and a new glacier inventory for Karakoram and Pamir region (https://www.earth-syst-sci-data-discuss.net/essd-2018-35/). The result reveals that our boundary performed better at higher elevations and covered 22% less area.

#### Glacier surface elevation

There were 5–8 stakes installed at each cross profile during the field survey in 1974–1975 by Batura Glacier Investigation Group (BGIG). The geodetic survey was conducted along the stakes. Data on glacier surface elevation, ice thickness, movement velocity, and ablation were acquired. The glacier surface elevation in 2017 was recorded by DGPS (SF-3050 GNSS, NavCom Technology, Inc., accuracy <0.05 m) on the way points of the ice thickness measurement. The surface elevation in 2000 was extracted from SRTM DEM (Shuttle Radar Topography Mission digital elevation model; February 11–22, 2000; http://srtm.csi.cgiar.org). ICESat satellite altimetry data for 2003–2008 were obtained from Kääb *et al*.^33^. The points of footprints within the main glacier part (cf. the sections B1 at 3200 m a.s.l., B2 at 3650 m a.s.l., and B3 at 4000 m a.s.l. in Fig. 1) were selected. Surface digital elevation models (DEMs) of the glacier under study were computed from freely available ASTER optical satellite stereo pairs for 2000–2016 by Brun *et al*.^32^. The calculated glacier surface elevation change rates (dh/dt)^32^ were used for the analysis.

### Ice thickness measurement

The gravimetric method was used by BGIG to estimate the ice thickness of Batura in 1974 (cf. red curves of P03, P06, P08, and P11 in Fig. 1). The thickness values were calculated from gravity observations with topographic, bouguer, and latitudinal correction (BGIG, 1976). Over 40 years later (in August 2017), the ice thickness at the same transverse cross-sections of Batura (P03, P06, and P08 in Fig. 1) was measured again by using GPR. An impulse radar system with separate transmitter and receiver, frequency centered at 5 MHz, antenna length of 10 m, and wavelength of 33.8 nm was used in this study. The equipment displayed good performance with high accuracy, which was verified through ice core drilling on the Hailuogou glacier of Gongga Mountain in Tibetan Plateau^55^. The transmitter and receiver, at a fixed distance of 5 m from each other, were carried on the glacier surface to measure the ice thickness at intervals of 50–200 m intervals along certain profiles. The time interval between the arrival of a wave directly through the air and that of its reflections from the glacier bed were used to calculate the ice thickness at the center point between the transmitter and receiver. The speed of electromagnetic wave propagation in ice is in the range 0.167–0.171 m ns^−1^ ^56–59^. Therefore, the wave speed was assumed to be 0.169 ± 0.002 m ns^−1^ in this study. The maximum error (for our highest thickness measurement, i.e., 201 m) is ±2.4 m. Another source of uncertainty is the accuracy of travel time, which was determined to be ± 10 ns (=±1.6 m) from the oscilloscope trace.

### Ice thickness calculation

Ice thickness was simulated by GlabTop2, which is a grid-based and slope-dependent estimation model^4,35^. The calculation requires a DEM and the glacier mask as input. The ice thickness is calculated for DEM cells selected automatically and randomly from the glacierized areas. Their distribution for all the glacier cells is then interpolated from the ice thickness at the randomly selected cells and at the glacier margins. Here, we set the depth of margin cells as 15 m. Detailed explanations of the model’s working and the determined of the model parameters have been provided by Frey *et al*.^4^. SRTM DEM with a spatial resolution of 3 arcsec (~90 m) was used as the surface elevation input in this study. The same was used by Frey *et al*. in the Himalayan–Karakoram region^4^. Hence, the difference in results could be attributed to the parameter selection. We executed the GlabTop2 model with multi parameterizations of basal shear stress *τ* ranging from 100 to 180 kPa and shape factor *f* ranging from 0.7 to 0.9. Given that the glacier erosion had occurred over a large timescale^60^, the glacier bed was assumed to have been relatively stable without tectonic activity during the past four decades. There is a 17 year difference between the model input data and GPR measurement. Therefore, the glacier bed position was used indirectly for model validation, rather than ice thickness. The optimal parameterization of minimum variance was selected to obtain the final ice thickness distribution. We observed that the maximum basal shear stress of 100 kPa was suitable for ice thickness calculation at P06 and P08 (both over 3000 m a.s.l.), where the debris was less. Meanwhile, 140 kPa was suitable for P03, which has a thick debris layer. Therefore, the basal shear stress was assumed to be decreasing gradually from 140 kPa at the glacier terminus to 100 kPa at 3000 m a.s.l.

### Mass conservation equation

For a certain portion of the Batura downstream between two flux gates (FGs), the equation of mass conservation^17^ states that the change in surface elevation (*h*) with time (*t*) between year 1 (*yr1*) and year 2 (*yr2*) is the sum of the area-average surface mass balance (*B*) and the flux term (all terms in m a^−1^ of ice):

1 $${\left\langle \frac{\delta h}{\delta t}\right\rangle }_{yr1-yr2}={\left\langle \frac{{\varphi }_{FGb}-{\varphi }_{FGf}}{A}\right\rangle }_{yr1-yr2}+\frac{{\left\langle B\right\rangle }_{yr1-yr2}}{\rho }$$

where *ρ* is the density of ice (900 kg m^−3^); ${\varphi }_{FGb}$ and ${\varphi }_{FGf}$ are the ice fluxes through the back and front FGs of each portion, respectively; and *A* is the glacier area between the flux gates. ${\langle \rangle }_{yr1-yr2}$ indicates the average between year 1 and year 2. ${\langle \frac{{\varphi }_{FGb}-{\varphi }_{FGf}}{A}\rangle }_{yr1-yr2}$ is the average emergence velocity (*v*_*e*_) of the flux portion between year 1 and year 2. The ice fluxes through each FG for two periods are estimated as follows:

2 $${\varphi }_{FG}={\int }_{s}Uds$$

where *s* represents the area of the cross-section and *U* is the component of ice velocity perpendicular to the cross-section. The mean cross-sectional velocities and cross-sectional areas are multiplied to compute the annual ice fluxes. The glacier bed topography from the model simulation is used to obtain the cross-sectional areas. The velocity values are available from two data sources. The annual surface velocities during 1974–1975 are calculated from the annual positioning of stakes anchored in the glacier. The centerline surface velocities of the subsequent period are extracted from the satellite-derived results^8^. Here, we used an intermediate value assuming that the depth-averaged velocity is 90% of the surface velocity^61^.

Consequently, to understand the change in the thinning rate (*Δh*, left-hand term in Eq. (1)) and surface mass balance (*B*, second right-hand term in Eq. (1)) of lower Batura from one period to another, we selected two periods (I:1974–1975 and II: 2007–2011) for comparison at four sections divided by three profiles. The emergence velocities of periods I and II were from the field survey by BGIG^27^ and satellite-derived velocity by Rankl *et al*.^8^, respectively. The surface mass balance (i.e., surface ablation) of the early period was acquired from stake observations of 1974–1975^27^. The surface thinning rates of the subsequent period were the average values from Brun *et al*.^32^. The other components of mass conservation (i.e., surface elevation change in period I and surface mass balance in period II) were computed according to Eq. (1).

### Uncertainties of mass balance

During the early period (1974–1975), the basal topography was within ±4.2% (of mean thickness)^27^ at each cross-section. The mean velocities were measured in the field with a precision of ±0.4 m a^−1^. After standard propagation of these errors in the ice flux equation^62^, the uncertainties vary between ±0.04 to ±0.09 m a^−1^ of ice. During the late period (2007–2011), the uncertainty of thinning rate is the area-average error of glacier surface elevation change rates ranging from ±0.49 to ±0.59 m a^−1^ of ice^32^. The error of calculated ice thickness imparts ±7.7 m of uncertainty (Fig. 2) to cross area calculation. The standard deviations of area-average surface velocities are ±8.41, ±4.14, and ±3.05 m a^−1^ for the portions of P11-08, P08-06, and P06-terminal, respectively. Therefore, using this calculation, the uncertainties of ice flux vary between ±0.40 and ±1.19 m a^−1^ of ice. After standard propagation of these errors, the total uncertainties of surface mass balance vary from ±1.29 m a^−1^ of ice at the high section to ±0.73 m a^−1^ of ice at the low section (Table 2).
